# Primary healthcare use prior to hospital consultations for spinal disorders among Danish children and adolescents: A descriptive nationwide registry-based study

**DOI:** 10.1007/s00431-026-07540-y

**Published:** 2026-10-29

**Authors:** Freja Gomez Overgaard, Michael Swain, Mette Wod, Lise Hestbæk, Henrik Hein Lauridsen, Søren Francis Dyhrberg O’Neill, Casper Nim

**Affiliations:** 1 Medical Spinal Research Unit, Spine Centre of Southern Denmark, University Hospital of Southern Denmark, Sygehusvej 24, 6000 Kolding, Denmark; 2 Department of Regional Health Research, University of Southern Denmark, Odense, Denmark; 3 Department of Chiropractic, Faculty of Medicine, Health and Human Science, Macquarie University, Sydney, Australia; 4 Center for Clinical Epidemiology, Odense University Hospital, Odense, Denmark; 5 Research Unit of Clinical Epidemiology, Department of Clinical Research, University of Southern Denmark, Odense, Denmark; 6 Center for Muscle and Joint Health, Department of Sports Science and Clinical Biomechanics, University of Southern Denmark, Odense, Denmark; 7 The Chiropractic Knowledge Hub, Odense, Denmark

**Keywords:** Pediatrics, Spinal diseases, Epidemiology, Diagnoses, Registries, Healthcare

## Abstract

Hospital-based spine-related diagnoses in children are often preceded by substantial healthcare use, but patterns and determinants remain poorly understood. This study aimed to describe pre-hospital primary healthcare use according to parental spine-related hospital diagnoses, the child’s comorbidity burden, and household income. We conducted a nationwide, registry-based cohort study including all Danish children aged 2–17 years with a first hospital-based spine-related diagnosis between 2009 and 2022 (*n* = 46,497). Healthcare services with general practitioners (GP), chiropractors, physiotherapists, and office-based specialist providers in the two years prior to diagnosis were quantified. Primary healthcare use was described across age groups, parental spine-related hospital diagnoses, the child’s comorbidity burden, and income tertiles. In the two years before diagnosis, children had a median of 9 reimbursed GP services (IQR [5-17]), 0 chiropractor services [0–0], 0 physiotherapy services [0–0], and 0 office-based specialist services [0–1]. GP use was common, whereas use of non-GP providers was highly skewed, with most children having no or few reimbursed services and a smaller subgroup having repeated service use. Descriptive patterns of healthcare use varied according to parental spine-related hospital diagnoses and comorbidity burden. Healthcare use showed no clear descriptive gradient across household income groups.

*Conclusion*: Patterns of primary healthcare use varied according to parental spine-related hospital diagnoses and comorbidity burden. No clear overall income gradient was observed, although patterns varied by provider type. These findings describe heterogeneous pre-hospital primary healthcare use in this population.

**Table:** 

**What is Known:** • *Spinal pain is common in children and is associated with substantial burden, including school absence and later risk of chronic pain.* • *Hospital-based spinal diagnoses in children are increasingly non-specific and often represent conditions that could be managed in primary care.*
**What is New:** • *Higher primary healthcare use was observed among children with greater comorbidity burden and among those with parental spine-related hospital diagnoses.* • *Differences in total primary healthcare use across household income groups were small, with no clear income gradient.*

**Supplementary information:**

The online version contains supplementary material available at https://doi.org/10.1007/s00431-026-07540-y.

## Introduction

Spinal pain in children and adolescents (hereafter referred to as children) is increasingly recognized as a significant public health concern, with up to 30% of school-aged children experiencing spinal pain within a year, and prevalence rates escalating during late adolescence [1, 2]. A Danish study identified five distinct growth trajectories of spinal pain frequency from ages 6 to 17, and while many children experience infrequent pain, a notable group of about 20% has increasing or persistent pain [3].

Spinal pain in childhood and adolescence is associated with consequences at the individual, family, and societal levels, contributing to the broader burden of musculoskeletal conditions, including increased healthcare utilization, school absenteeism, functional limitations, and psychosocial impact [4–6]. While many children experience transient or infrequent symptoms, it is the subgroup with persistent or recurrent spinal pain that accounts for a disproportionate share of this burden and healthcare engagement [4, 7]. Persistent spinal pain during adolescence is a strong predictor of spinal pain in adulthood [7, 8], adding to the life‑course societal burden and underscoring the importance of early identification and appropriate management.

Traditionally, spinal pain in children has been considered rare and primarily indicative of structural deformities such as scoliosis [9]. However, contemporary evidence suggests that most cases are nonspecific, similar to what is observed in adult spinal pain, necessitating a shift in clinical approach [10]. This is supported by a recent Danish registry-based study [11], which found that while the incidence rates of hospital-diagnosed spinal disorders remained stable over a 13-year period, the types of diagnoses shifted from structural (e.g., scoliosis) to more regional diagnoses (e.g., non-specific spinal pain).

These findings indicate that spinal disorders in children are often of a non-specific character, which, at least according to adult guidelines [12, 13], should be handled in the primary healthcare system. However, we know remarkably little about how these children present clinically or how they are handled in the primary healthcare system prior to hospital service. Factors such as parental spine-related hospital diagnoses, the presence of comorbidities, and socioeconomic status may influence healthcare-seeking behaviors [4, 5].

A more detailed understanding of these factors will offer insights to optimize the organization of health services. For example, it is not well understood to what extent children engage with primary healthcare before being assessed in hospital settings. This study, therefore, aims to describe all-cause pre-hospital primary healthcare use (general practitioners (GP), chiropractors, physiotherapists, and office-based specialist providers) two years before the hospital diagnosis, and examine how parental spine-related hospital diagnoses, comorbidities, household income, and diagnostic groups are related to pre-hospital healthcare use.

## Method

### Study design and setting

This nationwide, registry-based cohort study was conducted in Denmark using linked data from national health and administrative registries. The cohort definition and overall methodological approach follow the framework previously established [11], and key elements are summarized below. The study is reported according to the Strengthening the Reporting of Observational Studies in Epidemiology (STROBE) guidelines for observational studies [14].

According to Danish legislation, registry-based studies do not require formal ethical approval [15]. The project was registered with the Region of Southern Denmark (ID: 23/33311). Data access, management, and analyses were conducted on secure servers at Statistics Denmark, accessed via the research support unit OPEN at the Region of Southern Denmark [16].

### Data sources

Data were obtained from several Danish national registries which can be linked at the individual level using the unique personal identifier assigned to all residents of Denmark, the Civil Personal Registry number (CPR number). Data were obtained from:

- *The Danish Civil Registration System:* records vital demographic data for all Danish residents since 1968, including date of birth, sex, and links to biological parents via CPR numbers. It was used to obtain basic demographic data and to establish family linkages between children and parents [17].
- *The Danish National Patient Registry (DNPR):* contains information on all somatic hospital services, including inpatient, outpatient, and emergency visits. It includes dates of services, diagnostic information coded using International Classification of Diseases, 10th Revision (ICD-10), and hospital department codes. The DNPR was used to identify the study population of children diagnosed with spine-related conditions and to define comorbidities [11, 18].
- *The Income Statistics Registry:* was used to extract equivalized disposable family income at the child level [19].
- *The Population Registry*: provides information on country of origin and family structure (number of legal parents living with the child) [20].

### Study population

The study population included all children aged 2–17 years with a spine-related hospital diagnosis between 2009 and 2022 and no recorded spine-related hospital diagnosis during the two-year washout period (2007–2008) [11]. This approach identified hospital-based incident spinal diagnoses within the study period. The two-year observation period was selected a priori to capture healthcare utilization over a sufficiently long period to reflect broader pre-diagnostic healthcare patterns rather than short-term fluctuations immediately preceding diagnosis. Consequently, the cohort represents incident hospital-based diagnoses within the study period rather than lifetime incident cases, as diagnoses occurring before 2007 could not be excluded. We excluded infants (< 2 years) partly under the assumption that their healthcare patterns differ from older children with spine-related diagnoses, and partly because they could not contribute with two years of data. Spine-related diagnoses were identified using the ICD-10 [21] and included codes were: M4*, M5*, M96*, M99*, and S13*. These included both primary (A) and secondary (B) diagnosis codes. For each individual, the earliest recorded spine-related diagnosis was defined as the index diagnosis. The diagnostic codes were subsequently stratified into five clinical categories reflecting their potential severity and diagnostic domain: Critical (e.g., M46.4 spondylodiscitis, M80.4 vertebral osteonecrosis), Whiplash-related (e.g., S13.4 whiplash syndrome), Radiating (e.g., M47.9 spondylosis, M51.1 lumber disc herniation), Structural (e.g., M14.1 scoliosis), and Regional (e.g., M54.5 non-specific spinal pain). If multiple spinal diagnoses were recorded at the same index date, primary (A) codes were prioritized, and stratification was applied according to a predefined severity hierarchy, in which diagnoses were classified into mutually exclusive categories reflecting increasing clinical severity and each child was assigned to the highest-ranking category, as previously described [11].

The following exclusion criteria were applied: (1) missing linkage to parental or sociodemographic data, (2) invalid or replacement CPR numbers (e.g., codes for duplicate or annulled records), and (3) non-residency in Denmark at the index date or any part of the two-year pre-diagnostic period.

### Healthcare use

*Healthcare use* was defined as all-cause primary healthcare services recorded during the two years preceding the index diagnosis. For descriptive purposes, all-cause healthcare reimbursement services were summarized across the entire two-year period for each child and stratified by age group, parental spine-related hospital diagnoses, comorbidity burden, household income, and diagnostic group.

Healthcare use was quantified as the number of reimbursed primary healthcare services recorded in the national registries. These reimbursed services were based on the registry variable YDLANT, which records the number of services provided under each specialty. Because multiple reimbursed services may be recorded during a single clinical encounter, the measure does not necessarily represent unique patient visits, and coding practices may vary between provider types. For each child, all recorded reimbursement services were summed per provider type across the two‑year pre‑diagnostic period.

We identified services with: GPs, chiropractors, physiotherapists, and office-based specialist providers (e.g., pediatrics, neurology, orthopedics) [11]. Office-based specialist providers refer to office-based specialist physicians practicing outside hospitals within the Danish primary healthcare sector. These specialists are accessible through the National Health Service, typically following referral from a general practitioner, and therefore constitute part of primary healthcare in Denmark.

*Healthcare providers* were classified based on Danish registry specialty codes (SPEC2 and YDTYP), grouped into five categories as described in Appendix 1.

### Descriptive variables

#### Age groups

Age at the index diagnosis was categorized into three groups: 2–6 years, 7–12 years, and 13–17 years. These age groups are commonly defined by the WHO to reflect distinct developmental stages, i.e. early childhood, middle childhood, and adolescence [22].

#### Parental spine-related hospital diagnoses

Parental spine-related hospital diagnoses were identified in the DNPR using the same ICD-10 codes as for the child or adolescent (M4*, M5*, M96*, M99*, S13*). All available parental diagnostic records from 2007 onwards, up to the child’s index date were included. Parental spine-related hospital diagnoses were defined as whether no, one, or both parents had received a spine-related hospital diagnosis.

#### Child comorbidity

Comorbidity was defined as the number of distinct ICD-10 chapters represented in the DNPR in the two years prior to diagnosis. This chapter-based approach provides a proxy for multi-system disease burden and has previously been applied in pediatric populations where traditional comorbidity indices — such as the Charlson Comorbidity Index — are inadequate due to their heavy weighting toward adult chronic conditions and mortality risk [23]. In a pediatric cohort, the spectrum of disease burden is characterized by diverse multi-system or neurodevelopmental conditions rather than cumulative metabolic or cardiovascular decline. Thus, counting unique ICD-10 chapters was used as a pragmatic proxy for the breadth of healthcare-requiring conditions for the *breadth* of healthcare-requiring conditions, ensuring that a child or adolescent with diagnoses in both Chapter V (mental disorders) and Chapter X (respiratory diseases) would be considered to have a broader burden than one with diagnoses from only a single chapter. The burden was defined by the number of chapters present by diagnoses and categorized as: (1) No burden (0 chapters), (2) moderate burden (1–2 chapters), and (3) high burden (3 + chapters). Using ICD-10 chapters provides a measure of multimorbidity across different disease domains and reduces the influence of repeated coding of the same underlying condition.

#### Household income

Household income was captured for the index year on December 31 st and was used as a proxy for socioeconomic position during the preceding two-year observation period. Equivalized disposable household income was categorized into tertiles (low, medium, high) [24]. Household income tertiles were calculated separately for each calendar year within the study population and therefore reflect relative household income within the study population rather than the general population. To account for inflation and economic shifts over the full study period, these income tertiles were calculated within each separate calendar year, rather than pooling the data across the entire study duration. Equivalized in this context means that household income was adjusted for household size and composition according to the Organisation for Economic Co-operation and Development (OECD)-modified equivalence scale, where the first adult is assigned a weight of 1.0, each additional adult 0.5, and each child (< 14 years) 0.3, providing a standardized measure of economic resources per household member [19]. Participants with missing household income were retained in the cohort and categorized as “Unknown” in descriptive analyses to avoid excluding otherwise eligible participants.

#### Other study population characteristics used descriptively

- Sex was defined by a binary classification (female/male) as recorded in the national registry.
- Family composition classified as single-guardian or two-guardian/cohabiting household.
- Country of origin was defined according to the classification applied by Statistics Denmark, which uses a standard hierarchy when birthplace and citizenship do not concur [20]. We collapsed all non-Danish origins into “Other” due to limited data.

### Statistical analysis

The characteristics of the study population were summarized using counts (proportions) for categorical variables and medians with interquartile range (IQR) for continuous variables.

We summarized healthcare services by provider type (GP, chiropractor, physiotherapist, and office-based specialist providers) and by each descriptive variable using medians and interquartile ranges (IQR). The distributions were assessed descriptively using summary statistics and frequency distributions and were characterized by skewness and a large proportion of zero services, particularly for non-GP providers. Characteristics of the study cohort were descriptively compared with aggregated reference data from the general Danish pediatric population. Finally, since the aim of the study was descriptive, no statistical inference or modeling analyses were performed [25–27], nor was a formal comparison of groups or an estimate of effect sizes.

All analyses were conducted in R (version 4.3.0; R Core Team, 2023) using *Tidyverse* for data wrangling and illustrations and *gtsummary* for descriptive tables and outputs [28–30].

## Results

### Study population

We identified 46,497 children with a first-time hospital-based spine-related diagnosis between 2009 and 2022. The median age at diagnosis was 13.5 years (IQR 10.1–15.7), and 56% were female. Overall, the cohort predominantly comprised adolescents, children living in two-guardian households, and children of Danish origin. Additional characteristics of the study population, including diagnostic groups, parental spine-related diagnoses, comorbidity burden, household income, family composition, and country of origin, are presented in Table 1.

**Table 1:** Characteristics of the study sample

Characteristic		N = 46,497
Sex; n (%)		
	Female	25,908 (56%)
Age; median [IQR]		13.5 [10.1, 15.7]
Age groups; n (%)		
	2–6 years	5365 (12%)
	7–12 years	15,841 (34%)
	13–17 years	25,291 (54%)
Household income; median [IQR]		
	Lower third	146,546 [118,377–168,858]
	Middle third	225,735 [207,375–245,131]
	Upper third	332,066 [295,032–399,088]
	Unknown	(1.5%)
Family composition		
	Single parent	2803 (6%)
	Two parents	42,694 (94%)
Country of origin		
	Denmark	42,523 (91.5%)
	Other	3974 (8.5%)
Diagnoses groups; *n* (%)		
	Whiplash	17,543 (38%)
	Regional	13,881 (30%)
	Structural	13,596 (29%)
	Critical	882 (1.9%)
	Radiating	595 (1.3%)
Parents spinal diagnosis; *n* (%)		
	No parents	32,464 (70%)
	One parent	12,303 (26.3%)
	Two parents	1730 (3.7%)
Comorbidity chapters; *n* (%)		
	0	6410 (14%)
	1–2	16,138 (35%)
	3 +	23,949 (52%)

*N* = *total number in cohort, n* = *number reported for variable, IQR* = *interquartile range*

Household income was available for 98.5% of participants, with 1.5% categorized as “Unknown” due to missing income information.

#### Overall healthcare use in the two years before diagnosis

In the two years preceding the index diagnosis, children had a median of 9 GP reimbursement services [IQR 5–17]. Median reimbursement services were 0 [IQR 0–0] for chiropractors, 0 [IQR 0–0] for physiotherapists, and 0 [IQR 0–1] for office-based specialist providers. Across non-GP providers, the distributions were characterized by a large proportion of children with no recorded services, whereas a smaller subgroup had repeated service use. Detailed medians and IQRs for all providers and stratified subgroups are presented in Supplementary Table S1.

### Healthcare use by age group

**Fig. 1:**
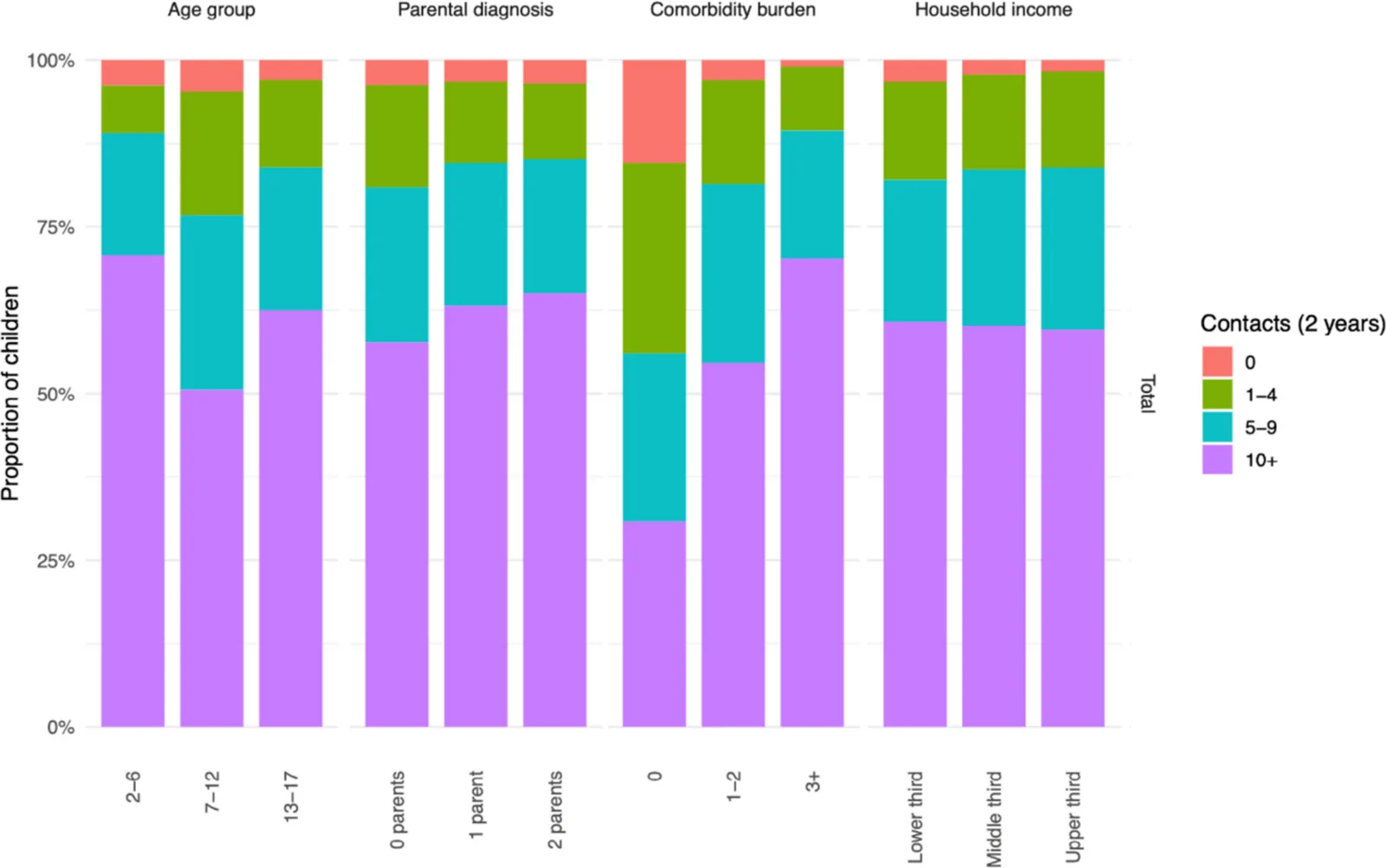
Total healthcare use (all providers combined) by age group, comorbidity burden, household income, and parental spine-related hospital diagnoses

**Fig. 2:**
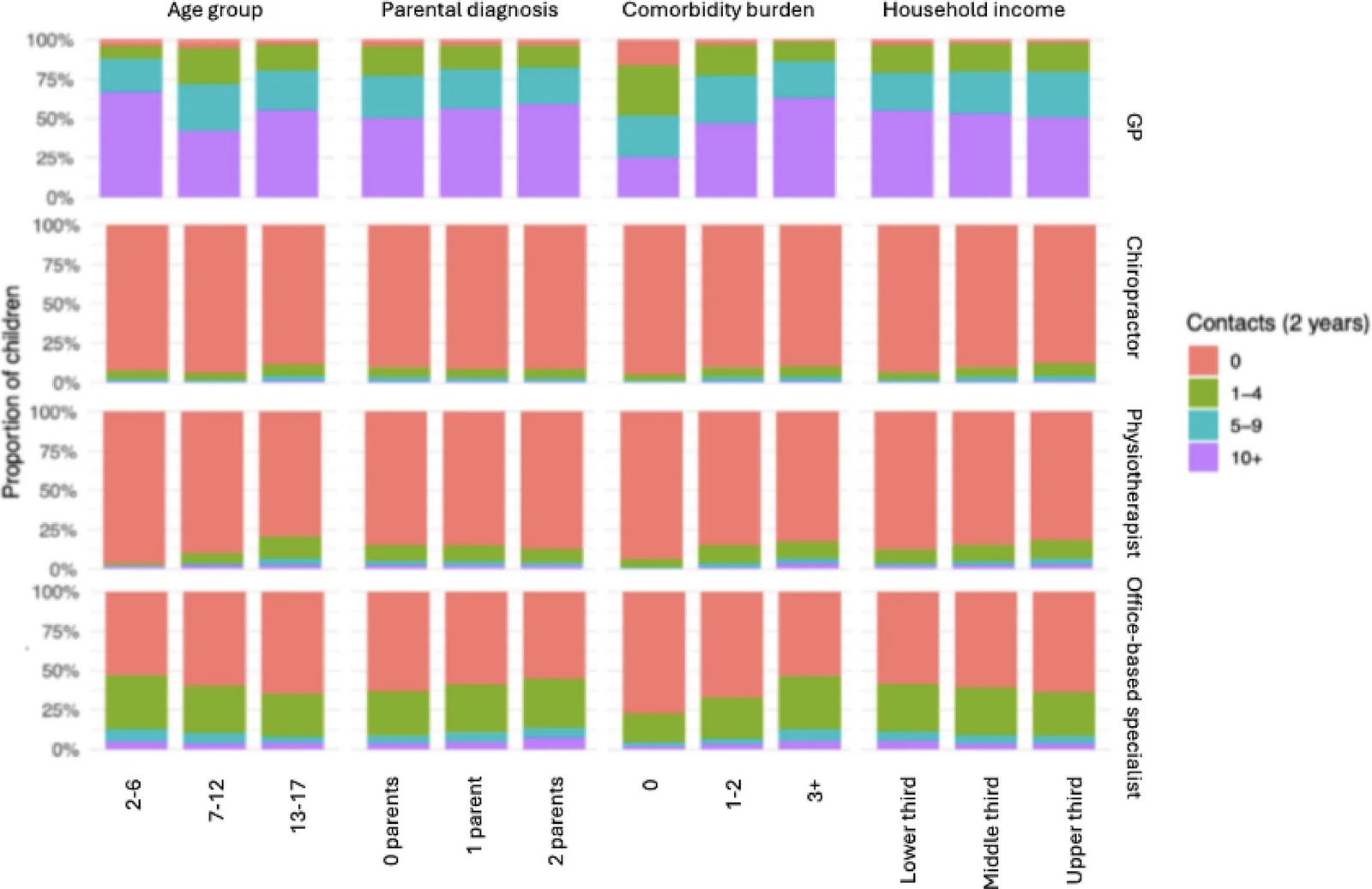
Distribution of healthcare use by provider type across age group, comorbidity burden, household income, and parental spine-related hospital diagnoses

Descriptive patterns of healthcare use varied across age groups and provider types. The 2–6-year age group had the highest median total healthcare use, primarily reflecting GP services (Fig. 1; Supplementary Table S1). Chiropractor and physiotherapist services were uncommon across all age groups, with the largest proportions of recorded use observed among children aged 13–17 years (Fig. 2). Medians and interquartile ranges by age group are shown in Supplementary Table S1.

### Healthcare use by parental spine-related hospital diagnoses

Descriptive patterns of healthcare use according to parental spine-related hospital diagnoses are presented in Figs. 1 and 2. The proportion of children in the higher service-use categories was largest among those with one or two affected parents, while chiropractor and physiotherapist services were uncommon across all parental diagnosis categories. Medians and interquartile ranges by parental spine-related hospital diagnoses are shown in Supplementary Table S1.

### Healthcare use by comorbidity

Descriptively, the proportion of children with ≥ 10 services was largest among those with greater comorbidity burden (Fig. 1). Among children with at least one comorbidity chapter, the most frequently represented ICD-10 chapters were Factors influencing health status and utilization with health services (ICD-10 chapter Z; 70.1%), Injury and poisoning (chapter S; 54.6%), Symptoms and abnormal clinical findings (chapter R; 23.6%), and External causes (chapters V and U; both 22.2%). Respiratory conditions (chapter J; 16.3%) and Injury, poisoning and external causes codes (chapter T; 12.8%) were also common. Musculoskeletal diagnoses excluding spinal diagnoses (chapter M) occurred in 11.8%, whereas Mental and behavioral disorders (chapter F) were recorded in 4.8%. A complete overview of chapter prevalence is presented in Supplementary Table S2.

When stratified by provider type, the largest differences across comorbidity categories were observed for GP services, while chiropractor services remained uncommon across all comorbidity categories (Fig. 2). Corresponding medians and interquartile ranges for healthcare use are shown in Supplementary Table S1.

### Healthcare use by income

No clear descriptive gradient in total healthcare use was observed across household income tertiles (Fig. 1). Provider-specific patterns varied across income tertiles (Fig. 2). Corresponding medians and interquartile ranges are shown in Supplementary Table S1.

### Healthcare use by diagnostic groups

Healthcare use across diagnostic groups is presented in Supplementary Table S3. Among children with radiating pain, 393 of 595 (66%) had ≥ 10 GP services. Frequent chiropractor and physiotherapist use was uncommon across diagnostic groups; among children with radiating pain, 17 (2.9%) had frequent chiropractor services and 22 (3.7%) had frequent physiotherapist services. Patterns of frequent office-based specialist provider use are also presented in Supplementary Table S3. The exact numbers and proportions underlying Figs. 1 and 2 are provided in Supplementary Table S4.

## Discussion

In this nationwide cohort of 46,497 Danish children with a first hospital-based spine-related diagnosis, healthcare use in the two years before diagnosis was heterogeneous. A two-year washout period was used to identify incident diagnoses; therefore, a small number of older children may have had hospital-based spine-related diagnoses before 2007 that could not be identified. GP services were the most common across all subgroups, whereas chiropractor, physiotherapist, and office-based specialist provider services were generally infrequent. Descriptive patterns of healthcare use varied according to parental spine-related hospital diagnoses, comorbidity burden, and age. Age-related patterns also differed by provider type, with the observed variation in overall healthcare use primarily reflecting GP services. These age-related patterns may reflect variation in healthcare needs and the increasing prevalence of musculoskeletal symptoms during adolescence. As the study captured all-cause healthcare utilization, the observed reimbursement services cannot be assumed to be directly related to spinal symptoms.

Importantly, this cohort included only children who received a first spine-related diagnosis in a hospital setting. In Denmark, access to hospital-based care typically requires referral from general practice or direct access through emergency departments. Consequently, this cohort likely represents children with more persistent or complex symptoms that were not fully managed in primary care, and the findings are therefore not generalizable to all children with spinal pain. While direct comparison with the general pediatric population is challenging due to differences in healthcare needs and routine services, the observed healthcare use is consistent with a population with greater healthcare needs beyond their spine-related diagnosis [5]. The observed median differences in all-cause healthcare use across provider types and patient subgroups may reflect differences in symptom persistence, comorbidity burden, and care pathways prior to hospital referral. Together, the observed healthcare utilization and comorbidity patterns suggest that many children in this cohort had complex healthcare needs extending beyond their spine-related condition. An additional explanation is that the present study captured only publicly reimbursed primary healthcare services. While this likely reflects the vast majority of GP services, some physiotherapy and chiropractic care is provided without public reimbursement and would therefore not be captured in the registries [32]. Consequently, the utilization of these provider groups may be underestimated.

To contextualize these findings, national data from the general Danish pediatric population indicate that in comparison with the general Danish pediatric population, the study cohort was older, with older children comprising a substantially larger proportion (54% aged 13–17 years vs. approximately 33% aged 13–17 years in the general population). The cohort also included a higher proportion of females (56% vs. approximately 49%) and a greater proportion of children living in two-guardian households (94% vs. approximately 80%). The proportion of children of Danish origin was similar to that of the general population (92% vs. approximately 89%) [31].

GP services were the most dominant form of healthcare use, likely reflecting the organization of the Danish healthcare system, where GPs function as the principal gatekeepers to hospital-based care and provide universally accessible first-line healthcare services. Although chiropractors may refer patients for selected diagnostic investigations, they do not have the same gatekeeping role as GPs for referral to secondary care. Consequently, referral from general practice is typically required before hospital assessment, and repeated GP use may reflect ongoing symptom management, monitoring, or referral processes prior to hospital diagnosis. In contrast, chiropractor and physiotherapist use were generally infrequent across subgroups partly reflecting differences in access and organization of care across provider types. GP care is free of charge in Denmark, whereas chiropractor and physiotherapist care involve out-of-pocket payment. Descriptive patterns of provider-specific healthcare use also varied across household income groups, although no clear gradient was observed for total healthcare use. The substantially greater use of GP services than other provider types should also be interpreted in the context of the organization of the Danish healthcare system. GPs serve as the primary point of contact and gatekeepers to much of specialist and hospital care, meaning that children requiring further assessment or referral will commonly encounter a GP as part of their care pathway. Differences in healthcare use across provider types therefore partly reflect differences in their roles and accessibility within the healthcare system and should not be interpreted as direct comparisons of healthcare need across provider types.

The low level of chiropractor services observed in this study contrasts with higher utilization reported in other Danish populations, such as adults and infants [33], and may reflect that chiropractor care is generally less commonly utilized among children in Denmark [31], rather than being specific to the pre-diagnostic pathway in this cohort. It might also reflect that children seeking chiropractic or physiotherapy care are adequately managed in primary care. However, the underlying reasons for these patterns cannot be determined from the present data.

The descriptive pattern of healthcare use across comorbidity categories may reflect differences in healthcare needs, as described in pediatric populations with multisystem conditions [5, 34]. Among children with at least one comorbidity chapter, diagnoses related to healthcare services, injuries, symptoms, and respiratory conditions were common, indicating that diagnoses recorded during the observation period were not limited to musculoskeletal conditions. Surveillance bias may also contribute to this finding, as children with greater healthcare needs are more likely to interact with healthcare providers, increasing the opportunity for hospital referral and diagnosis. Furthermore, the available registry data did not contain information on the clinical indication or diagnoses associated with individual primary healthcare use and therefore did not allow assessment of delayed or missed diagnoses.

The descriptive patterns observed according to parental spine-related hospital diagnoses may reflect shared genetic or environmental factors, as well as learned healthcare-seeking behaviors within families [35, 36]. However, parental spine-related hospital diagnoses in the present study were based on hospital registry data, and therefore likely reflect a subgroup of parents with more severe or persistent spinal conditions.

Although alternative observation windows could be considered, the purpose of the present study was to provide a descriptive overview of healthcare utilization during the two years preceding hospital diagnosis, which was selected a priori to capture longer-term healthcare trajectories.

The present study describes all-cause healthcare use prior to hospital-based spinal diagnoses, but the reasons for individual healthcare use were not available. Future research should investigate spine-specific healthcare use, referral pathways, and trajectories leading to hospital assessment among children with spinal pain, but also, more qualitative approaches would seem sufficient to describe care-seeking behaviors.

### Strengths and limitations

This study has several strengths. The nationwide design, based on linked Danish registries, enabled inclusion of all children with a hospital-based spine-related diagnosis over a 13-year period, minimizing selection bias and ensuring high external validity within the defined population. Individual-level linkage to parents and socioeconomic data allowed consistent characterization of family context with minimal missing data. Furthermore, the Danish National Patient Registry is known for high completeness and validity [18], supporting robust ascertainment of hospital-based diagnoses. However, registry “services” reflect reimbursed procedures rather than unique visits, which may overstate discrete encounters. Because a single clinical encounter may generate multiple reimbursement codes, and coding practices may vary across provider types, differences in healthcare use may partly reflect variation in coding behavior rather than true differences in visit frequency.

Some limitations should be considered. First, the hospital-based case definition limits generalizability to children referred to secondary care, and findings do not capture those managed exclusively in primary care. Second, registry data captured all-cause healthcare use, and the reasons for individual services were not available. Consequently, healthcare services cannot be assumed to be directly related to spinal symptoms. Third, the chapter-based comorbidity measure was used as a pragmatic proxy and has not been specifically validated as a measure of disease burden in this population. In addition, ICD-10 chapter Z includes administrative and non-procedural codes that do not necessarily reflect comorbidity, introducing a potential risk of misclassification. Fourth, as no inferential statistical comparisons were performed, observed differences between subgroups should be interpreted descriptively and not as evidence of statistically supported group differences. Lastly, household income was measured for the index year and may therefore not fully reflect socioeconomic circumstances throughout the entire two-year pre-diagnostic period.

## Conclusion

Danish children with a first hospital-based spine-related diagnosis are clinically diverse and have heterogeneous patterns of healthcare use in the two years before diagnosis. Higher healthcare use was observed among children with parental spine-related hospital diagnoses and greater comorbidity burden. However, most children had few or no reimbursement services with physiotherapists, chiropractors, and office-based specialist providers.

## Supplementary information

Below is the link to the electronic supplementary material.

Supplementary file1 (DOCX 36 kb)

Supplementary file2 (DOCX 17 kb)

Supplementary file3 (DOCX 18 kb)

Supplementary file4 (DOCX 21 kb)

Supplementary file5 (PDF 203 kb)

## Data Availability

No datasets were generated or analysed during the current study.

## Abbreviations

CPR: Civil Personal Registry
CRS: Civil Registration System
DNPR: Danish National Patient Registry
GP: General practitioner
ICD-10: International Classification of Diseases, 10th Revision
IQR: Interquartile range
SD: Standard deviation
STROBE: Strengthening the Reporting of Observational Studies in Epidemiology
