# Supplementary material for: Primary healthcare use prior to hospital consultations for spinal disorders among Danish children and adolescents: A descriptive nationwide registry-based study
Source: Eur J Pediatr. 2026 Oct 29;185(11):903. doi: 10.1007/s00431-026-07540-y (PMC13719717; doi:10.1007/s00431-026-07540-y)
Supplement: Supplementary file 1 [file 431_2026_7540_MOESM5_ESM.pdf]

Supplementary Table S4. Distribution of reimbursed primary healthcare services in the two years before diagnosis, by descriptive subgroup and provider type.

**Characteristic**	**Level**	**Provider**	**0 services**	**1-4 services**	**5-9 services**	**>= 10 services**
Age group	2-6	Total healthcare use	213 (3.8%)	421 (7.4%)	1071 (18.9%)	3957 (69.9%)
Age group	7-12	Total healthcare use	813 (4.9%)	3203 (19.2%)	4375 (26.3%)	8270 (49.6%)
Age group	13-17	Total healthcare use	811 (3%)	3670 (13.8%)	5810 (21.8%)	16396 (61.4%)
Age group	2-6	GP	218 (3.9%)	491 (8.7%)	1232 (21.8%)	3721 (65.7%)
Age group	7-12	GP	931 (5.6%)	3909 (23.5%)	4938 (29.6%)	6993 (41.3%)
Age group	13-17	GP	895 (3.4%)	4425 (16.6%)	6854 (25.7%)	14513 (54.4%)
Age group	2-6	Chiropractor	5240 (92.5%)	294 (5.2%)	95 (1.7%)	33 (0.6%)
Age group	7-12	Chiropractor	15633 (93.8%)	727 (4.4%)	233 (1.4%)	68 (0.4%)
Age group	13-17	Chiropractor	23682 (88.7%)	1891 (7.1%)	840 (3.1%)	274 (1%)
Age group	2-6	Physiotherapist	5472 (96.6%)	81 (1.4%)	16 (0.3%)	93 (1.6%)
Age group	7-12	Physiotherapist	15021 (90.2%)	1063 (6.4%)	192 (1.2%)	385 (2.3%)
Age group	13-17	Physiotherapist	21360 (80%)	3679 (13.8%)	1019 (3.8%)	629 (2.4%)
Age group	2-6	Office-based specialist provider	3022 (53.4%)	1923 (34%)	443 (7.8%)	274 (4.8%)
Age group	7-12	Office-based specialist provider	10018 (60.1%)	4937 (29.6%)	1090 (6.5%)	616 (3.7%)
Age group	13-17	Office-based specialist provider	17310 (64.9%)	7220 (27.1%)	1045 (3.9%)	1112 (4.2%)
Parental spine-related hospital diagnosis	0 parents	Total healthcare use	1369 (3.9%)	5600 (16%)	8282 (23.7%)	19726 (56.4%)
Parental spine-related hospital diagnosis	1 parent	Total healthcare use	406 (3.3%)	1499 (12.2%)	2626 (21.3%)	7772 (63.2%)
Parental spine-related hospital diagnosis	2 parents	Total healthcare use	62 (3.6%)	195 (11.3%)	384 (20.1%)	1125 (65%)
Parental spine-related hospital diagnosis	0 parents	GP	1517 (4.3%)	6772 (19.4%)	9529 (27.2%)	17159 (49.1%)
Parental spine-related hospital diagnosis	1 parent	GP	462 (3.8%)	1814 (14.7%)	3091 (25.1%)	6936 (56.4%)
Parental spine-related hospital diagnosis	2 parents	GP	65 (3.8%)	239 (13.8%)	404 (23.4%)	1022 (59.1%)
Parental spine-related hospital diagnosis	0 parents	Chiropractor	31740 (90.7%)	2107 (6%)	865 (2.5%)	265 (0.8%)
Parental spine-related hospital diagnosis	1 parent	Chiropractor	11237 (91.3%)	700 (5.7%)	268 (2.2%)	98 (0.8%)
Parental spine-related hospital diagnosis	2 parents	Chiropractor	1578 (91.2%)	105 (6.1%)	35 (2%)	12 (0.7%)
Parental spine-related hospital diagnosis	0 parents	Physiotherapist	29855 (85.4%)	3439 (9.8%)	863 (2.5%)	820 (2.3%)
Parental spine-related hospital diagnosis	1 parent	Physiotherapist	10483 (85.2%)	1237 (10.1%)	330 (2.7%)	253 (2.1%)
Parental spine-related hospital diagnosis	2 parents	Physiotherapist	1515 (87.6%)	147 (8.5%)	34 (2%)	34 (2%)
Parental spine-related hospital diagnosis	0 parents	Office-based specialist provider	22172 (63.4%)	9774 (27.9%)	1731 (4.9%)	1300 (3.7%)
Parental spine-related hospital diagnosis	1 parent	Office-based specialist provider	7226 (58.7%)	3764 (30.6%)	740 (6%)	573 (4.7%)
Parental spine-related hospital diagnosis	2 parents	Office-based specialist provider	952 (55%)	542 (31.3%)	107 (6.2%)	129 (7.5%)
Comorbidity burden	0	Total healthcare use	1051 (15.3%)	2019 (29.3%)	1741 (25.3%)	2080 (30.2%)
Comorbidity burden	1-2	Total healthcare use	533 (3.1%)	2835 (16.4%)	4712 (27.2%)	9223 (53.3%)
Comorbidity burden	3+	Total healthcare use	253 (1%)	2440 (9.8%)	4803 (19.4%)	17320 (69.8%)
Comorbidity burden	0	GP	1100 (16%)	2250 (32.7%)	1835 (26.6%)	1706 (24.8%)
Comorbidity burden	1-2	GP	609 (3.5%)	3471 (20.1%)	5318 (30.7%)	7905 (45.7%)
Comorbidity burden	3+	GP	335 (1.3%)	3104 (12.5%)	5871 (23.7%)	15506 (62.5%)
Comorbidity burden	0	Chiropractor	6548 (95%)	247 (3.6%)	79 (1.1%)	17 (0.2%)
Comorbidity burden	1-2	Chiropractor	15704 (90.8%)	1035 (6%)	430 (2.5%)	134 (0.8%)
Comorbidity burden	3+	Chiropractor	22303 (89.9%)	1630 (6.6%)	659 (2.7%)	224 (0.9%)
Comorbidity burden	0	Physiotherapist	6486 (94.1%)	330 (4.8%)	61 (0.9%)	14 (0.2%)
Comorbidity burden	1-2	Physiotherapist	14787 (85.5%)	1896 (11%)	441 (2.5%)	179 (1%)
Comorbidity burden	3+	Physiotherapist	20580 (82.9%)	2597 (10.5%)	725 (2.9%)	914 (3.7%)
Comorbidity burden	0	Office-based specialist provider	5354 (77.7%)	1240 (18%)	151 (2.2%)	146 (2.1%)
Comorbidity burden	1-2	Office-based specialist provider	11650 (67.3%)	4515 (26.1%)	583 (3.4%)	555 (3.2%)
Comorbidity burden	3+	Office-based specialist provider	13346 (53.8%)	8325 (33.5%)	1844 (7.4%)	1301 (5.2%)
Household income	Lower third	Total healthcare use	554 (3.4%)	2410 (15%)	3455 (21.5%)	9687 (60.1%)
Household income	Middle third	Total healthcare use	358 (2.2%)	2376 (14.8%)	3796 (23.6%)	9575 (59.5%)
Household income	Upper third	Total healthcare use	304 (1.9%)	2490 (15.5%)	3989 (24.8%)	9323 (57.9%)
Household income	Lower third	GP	608 (3.8%)	2848 (17.7%)	3893 (24.2%)	8757 (54.4%)
Household income	Middle third	GP	431 (2.7%)	2889 (17.9%)	4358 (27.1%)	8427 (52.3%)
Household income	Upper third	GP	384 (2.4%)	3067 (19%)	4758 (29.5%)	7897 (49%)
Household income	Lower third	Chiropractor	15070 (93.6%)	703 (4.4%)	255 (1.6%)	78 (0.5%)
Household income	Middle third	Chiropractor	14579 (90.5%)	979 (6.1%)	412 (2.6%)	135 (0.8%)
Household income	Upper third	Chiropractor	14217 (88.3%)	1226 (7.6%)	501 (3.1%)	162 (1%)
Household income	Lower third	Physiotherapist	14181 (88%)	1341 (8.3%)	281 (1.7%)	303 (1.9%)
Household income	Middle third	Physiotherapist	13654 (84.8%)	1666 (10.3%)	431 (2.7%)	354 (2.2%)
Household income	Upper third	Physiotherapist	13337 (82.8%)	1814 (11.3%)	513 (3.2%)	442 (2.7%)
Household income	Lower third	Office-based specialist provider	9466 (58.8%)	4865 (30.2%)	911 (5.7%)	864 (5.4%)
Household income	Middle third	Office-based specialist provider	9825 (61%)	4858 (30.2%)	858 (5.3%)	564 (3.5%)
Household income	Upper third	Office-based specialist provider	10394 (64.5%)	4333 (26.9%)	806 (5%)	573 (3.6%)

Values are presented as n (%). Service-use categories correspond to those used in Figures 1 and 2.
