# Supplementary material for: Primary healthcare use prior to hospital consultations for spinal disorders among Danish children and adolescents: A descriptive nationwide registry-based study
Source: Eur J Pediatr. 2026 Oct 29;185(11):903. doi: 10.1007/s00431-026-07540-y (PMC13719717; doi:10.1007/s00431-026-07540-y)
Supplement: Supplementary file 2 [file 431_2026_7540_MOESM1_ESM.docx]

Healthcare provider

SPEC2 and YDTYP

Divided into 5 groups

| **SPEC2** | | |
| --- | --- | --- |
| **Number** | **Original** | **Group** |
| 00 | Not specified | Other |
| 01 | Anesthesiology | Office-based specialist provider |
| 02 | Fictional yield limit | Other |
| 03 | Diagnostically radiology Copenhagen | Office-based specialist provider |
| 04 | Dermato-venerology | Office-based specialist provider |
| 05 | Diagnostically radiology | Office-based specialist provider |
| 06 | Rheumatology | Office-based specialist provider |
| 07 | Gynecology/obstetric | Office-based specialist provider |
| 08 | Internal medicine | Office-based specialist provider |
| 09 | Surgery | Office-based specialist provider |
| 10 | Histopathological examinations | Office-based specialist provider |
| 11 | Clinical chemistry | Office-based specialist provider |
| 15 | ENT emergency service Copenhagen | Office-based specialist provider |
| 16 | ENT emergency service KAK | Office-based specialist provider |
| 17 | Neurosurgery | Office-based specialist provider |
| 18 | Neuromedicine | Office-based specialist provider |
| 19 | Opthalmologist emergency service | Office-based specialist provider |
| 20 | Orthopedic surgery | Office-based specialist provider |
| 21 | ENT emergency service | Office-based specialist provider |
| 22 | Pathology | Office-based specialist provider |
| 23 | Plastic surgery | Office-based specialist provider |
| 24 | Psychiatry | Office-based specialist provider |
| 25 | Pediatrics | Office-based specialist provider |
| 26 | Child Psychiatry | Office-based specialist provider |
| 28 | Tropical medicine | Office-based specialist provider |
| 35 | District psychiatry | Office-based specialist provider |
| 39 | Ophthalmologist assistance kfa | Office-based specialist provider |
| 41 | ENT assistance kfa | Office-based specialist provider |
| 42 | Reference Laboratory | Other |
| 43 | Diagnostic laboratory | Other |
| 44 | Copenhagen general practitioners’ laboratory | Other |
| 45 | Medical laboratory | Other |
| 46 | Regional laboratories | Other |
| 47 | University of Aarhus | Other |
| 48 | Statens Serum Institute | Other |
| 49 | Dental hygienists | Other |
| 50 | Dental care | Other |
| 51 | Physiotherapy 51 | Physiotherapy |
| 52 | Glasses | Other |
| 53 | Chiropractic | Chiropractic |
| 54 | Podiatry | Other |
| 55 | Orthonyxia | Other |
| 56 | Clinic treatment | Other |
| 57 | Equine-assisted therapy | Physiotherapy |
| 58 | Rehabilitation, Øfeldt | Physiotherapy |
| 59 | Podiatry for scar tissue | Other |
| 60 | Podiatry for rheumatoid arthritis | Other |
| 61 | Physiotherapy 61 | Physiotherapy |
| 62 | Free physiotherapy | Physiotherapy |
| 63 | Psychology | Office-based specialist provider |
| 64 | Chiropractic for chronic | Chiropractic |
| 65 | Free equine-assisted therapy | Physiotherapy |
| 68 | Hearing care | Other |
| 70 | General practitioner, day consultation | General Practitioner |
| 71 | General practitioner, evening consultation | General Practitioner |
| 72 | General practitioner, day phone consultation | General Practitioner |
| 73 | General practitioner, evening phone consultation | General Practitioner |
| 74 | General practitioner, day visit | General Practitioner |
| 75 | General practitioner, evening visit | General Practitioner |
| 76 | General practitioner, email consultation | General Practitioner |
| 77 | General practitioner, other services | General Practitioner |
| 78 | General practitioner, contact prevention | General Practitioner |
| 79 | General practitioner, basis | General Practitioner |
| 80 | General practitioner assistance 80 | General Practitioner |
| 81 | KFA on-call service 81 | General Practitioner |
| 82 | Kak on-call doctor assistance 82 | Other |
| 83 | On-call doctor assistance 83 | Other |
| 84 | Kak on-call doctor assistance 84 | Other |
| 85 | Pre-hospital | Other |
| 86 | Island doctors | Other |
| 87 | Screening, sexually transmitted diseases | Other |
| 88 | Non-contracted doctor | General Practitioner |
| 89 | KFA on-call service 89 | Other |
| 90 | Public health insurance | Other |
| 91 | Exemption from health services | Other |
| 92 | Prescription medications, etc. | General Practitioner |
| 93 | Referral to specialist | Office-based specialist provider |
| 94 | Subsidy for nutritional supplements | Other |
| 95 | Funeral service | Other |
| 96 | Translation assistance | Other |
| 97 | Funding | Other |
| 98 | Consultation by general practitioners | General Practitioner |
| 99 | Miscellaneous services | Other |

| **YDTYP** | | |
| --- | --- | --- |
| **Number** | **Original** | **Group** |
| 00 | Not specified | Other |
| 01 | General Practitioner | General Practitioner |
| 02 | General Practitioner | General Practitioner |
| 03 | General Practitioner | General Practitioner |
| 04 | General Practitioner | General Practitioner |
| 05 | General Practitioner | General Practitioner |
| 06 | On-call doctor | Other |
| 07 | On-call doctor | Other |
| 08 | On-call doctor | Other |
| 09 | On-call doctor | Other |
| 10 | On-call doctor | Other |
| 11 | Ophtalmologist | Other |
| 12 | Ophtalmologist | Other |
| 13 | Ophtalmologist | Other |
| 14 | Ophtalmologist | Other |
| 15 | Ophtalmologist | Other |
| 16 | ENT doctor | Office-based specialist provider |
| 17 | ENT doctor | Office-based specialist provider |
| 18 | ENT doctor | Office-based specialist provider |
| 19 | ENT doctor | Office-based specialist provider |
| 20 | ENT doctor | Office-based specialist provider |
| 22 | Other medical specialist | Office-based specialist provider |
| 23 | Other medical specialist | Office-based specialist provider |
| 24 | Other medical specialist | Office-based specialist provider |
| 25 | Other medical specialist | Office-based specialist provider |
| 26 | Other medical specialist | Office-based specialist provider |
| 27 | Other medical specialist | Office-based specialist provider |
| 28 | Other medical specialist | Office-based specialist provider |
| 29 | Other medical specialist | Office-based specialist provider |
| 30 | Other medical specialist | Office-based specialist provider |
| 31 | Other medical specialist | Office-based specialist provider |
| 32 | Other medical specialist | Office-based specialist provider |
| 33 | Other medical specialist | Office-based specialist provider |
| 34 | Other medical specialist | Office-based specialist provider |
| 35 | Other medical specialist | Office-based specialist provider |
| 38 | Dentist | Other |
| 39 | Dentist | Other |
| 40 | Dentist | Other |
| 41 | Physiurgical clinic | Other |
| 42 | Riding school | Physiotherapy |
| 43 | Physiotherapist | Physiotherapy |
| 44 | Physiotherapist | Physiotherapy |
| 45 | Physiotherapist | Physiotherapy |
| 46 | Teddy Øfeldt | Physiotherapy |
| 48 | Chiropractor | Chiropractic |
| 49 | Chiropractor | Chiropractic |
| 50 | Chiropractor | Chiropractic |
| 52 | Optician | Other |
| 53 | Optician | Other |
| 54 | Optician | Other |
| 55 | Optician | Other |
| 57 | Podiatrist | Other |
| 58 | Podiatrist | Other |
| 59 | Podiatrist | Other |
| 60 | Podiatrist | Other |
| 64 | Pharmacy | Other |
| 65 | Pharmacy | Other |
| 66 | Laboratory | Other |
| 67 | Laboratory | Other |
| 68 | Laboratory | Other |
| 69 | Laboratory | Other |
| 70 | Laboratory | Other |
| 71 | Hospital | Office-based specialist provider |
| 72 | Municipalities/counties | Other |
| 73 | Municipalities/counties | Other |
| 74 | Municipalities/counties | Other |
| 75 | Municipalities/counties | Other |
| 76 | Municipalities/counties | Other |
| 77 | Foundations | Other |
| 78 | Foundations | Other |
| 79 | Foundations | Other |
| 80 | House doctor | General Practitioner |
| 81 | On-call managers | Other |
| 88 | Hearing aids | Other |
| 90 | Fictional | Other |
| 91 | Funeral directors | Other |
| 92 | Non existing | Other |
| 93 | Psychologist | Office-based specialist provider |
| 94 | Psychologist | Office-based specialist provider |
| 95 | Nursing supplies | Other |
| 96 | Nutritional supplements | Other |
| 97 | Translator | Other |
| 98 | Haulier | Other |
| 99 | Falck rescue services | Other |

Note: Office-based specialist providers include provider types considered clinically relevant to the management of children with spine-related conditions. Other office-based specialties (e.g., ophthalmology) were classified as "Other" and were not included in the specialist provider category.
