# Supplementary material for: Primary healthcare use prior to hospital consultations for spinal disorders among Danish children and adolescents: A descriptive nationwide registry-based study
Source: Eur J Pediatr. 2026 Oct 29;185(11):903. doi: 10.1007/s00431-026-07540-y (PMC13719717; doi:10.1007/s00431-026-07540-y)
Supplement: Supplementary file 3 [file 431_2026_7540_MOESM2_ESM.docx]

Supplementary Table S1. Median and interquartile range (IQR) of healthcare contacts in the two years before diagnosis, stratified by age group, parental spine diagnosis, comorbidity burden, and household income.

| Exposure | Level | N | Total | GP | Chiropractor | Physiotherapist | Medical specialist |
| --- | --- | --- | --- | --- | --- | --- | --- |
| Age group | 2-6 | 5,365 | 15 [8-25] | 13 [8-21] | 0 [0-0] | 0 [0-0] | 0 [0-2] |
| Age group | 7-12 | 15,841 | 10 [5-18] | 8 [4-14] | 0 [0-0] | 0 [0-0] | 0 [0-2] |
| Age group | 13-17 | 25,291 | 13 [7-24] | 11 [6-19] | 0 [0-0] | 0 [0-0] | 0 [0-1] |
| Comorbidity chapters | 0 | 6,410 | 5 [2-11] | 5 [2-10] | 0 [0-0] | 0 [0-0] | 0 [0-0] |
| Comorbidity chapters | 1-2 | 16,138 | 11 [6-18] | 9 [5-15] | 0 [0-0] | 0 [0-0] | 0 [0-1] |
| Comorbidity chapters | 3+ | 23,949 | 15 [8-27] | 13 [7-21] | 0 [0-0] | 0 [0-0] | 0 [0-2] |
| Household income | Lower third | 15,270 | 13 [6-24] | 11 [5-19] | 0 [0-0] | 0 [0-0] | 0 [0-2] |
| Household income | Middle third | 15,270 | 12 [6-22] | 10 [5-18] | 0 [0-0] | 0 [0-0] | 0 [0-2] |
| Household income | Upper third | 15,270 | 12 [6-21] | 10 [5-16] | 0 [0-0] | 0 [0-0] | 0 [0-1] |
| Household income | Unknown | 688 (1.5%) | - | - | - | - | - |
| Parental spine diagnosis | 0 parents | 32,464 | 11 [6-21] | 10 [5-17] | 0 [0-0] | 0 [0-0] | 0 [0-1] |
| Parental spine diagnosis | 1 parent | 12,303 | 13 [7-24] | 11 [6-19] | 0 [0-0] | 0 [0-0] | 0 [0-2] |
| Parental spine diagnosis | 2 parents | 1,730 | 14 [7-26] | 12 [6-21] | 0 [0-0] | 0 [0-0] | 0 [0-2] |

Note: Values are presented as *median [interquartile range]*. A “contact” refers to any reimbursed primary care service recorded in national registries; multiple contacts may occur within a single clinical visit.
