# Supplementary material for: Primary healthcare use prior to hospital consultations for spinal disorders among Danish children and adolescents: A descriptive nationwide registry-based study
Source: Eur J Pediatr. 2026 Oct 29;185(11):903. doi: 10.1007/s00431-026-07540-y (PMC13719717; doi:10.1007/s00431-026-07540-y)
Supplement: Supplementary file 4 [file 431_2026_7540_MOESM3_ESM.docx]

Supplementary Table S2. Distribution of ICD-10 diagnostic chapters among children in the study population

| **ICD-10 Chapter name** | **ICD-10 Chapter** | **N children** | **%** |
| --- | --- | --- | --- |
| Factors influencing health status and contact with health services | Z | 36,541 | 70.1 |
| Injury, poisoning and certain other consequences of external causes | S | 28,473 | 54.6 |
| Symptoms, signs and abnormal clinical and laboratory findings, not elsewhere classified | R | 12,290 | 23.6 |
| External causes of morbidity and mortality | V | 11,559 | 22.2 |
| Codes for special purposes | U | 11,558 | 22.2 |
| Diseases of the respiratory system | J | 8,502 | 16.3 |
| Injury, poisoning and certain other consequences of external causes | T | 6,657 | 12.8 |
| Diseases of the musculoskeletal system and connective tissue | M | 6,169 | 11.8 |
| Diseases of the digestive system | K | 5,397 | 10.4 |
| Diseases of the ear, mastoid process, eye and adnexa | H | 5,234 | 10.0 |
| Congenital malformations, deformations and chromosomal abnormalities | Q | 5,024 | 9.6 |
| Certain conditions originating in the perinatal period | P | 4,122 | 7.9 |
| Diseases of the genitourinary system | N | 3,148 | 6.0 |
| Endocrine, nutritional and metabolic diseases | E | 3,009 | 5.8 |
| Certain infectious and parasitic diseases | B | 2,980 | 5.7 |
| Diseases of the skin and subcutaneous tissue | L | 2,754 | 5.3 |
| Diseases of the nervous system | G | 2,720 | 5.2 |
| Certain infectious and parasitic diseases | A | 2,693 | 5.2 |
| Mental and behavioural disorders | F | 2,483 | 4.8 |
| Diseases of the blood and blood-forming organs and certain disorders involving the immune mechanism | D | 1,413 | 2.7 |
| Diseases of the circulatory system | I | 744 | 1.4 |
| Pregnancy, childbirth and the puerperium | O | 160 | 0.3 |
| Neoplasms | C | 124 | 0.2 |
| External causes of morbidity and mortality | Y | 99 | 0.2 |
| External causes of morbidity and mortality | X | 46 | 0.1 |

Note: Values represent the number and percentage of children with at least one diagnosis within each ICD-10 chapter in the study period. Children may contribute to multiple chapters, and percentages therefore do not sum to 100%. ICD-10 chapter codes are presented alongside chapter names.
