# Supplementary material for: Primary healthcare use prior to hospital consultations for spinal disorders among Danish children and adolescents: A descriptive nationwide registry-based study
Source: Eur J Pediatr. 2026 Oct 29;185(11):903. doi: 10.1007/s00431-026-07540-y (PMC13719717; doi:10.1007/s00431-026-07540-y)
Supplement: Supplementary file 5 [file 431_2026_7540_MOESM4_ESM.docx]

Supplementary Table S3. Distribution of healthcare contacts by provider and diagnosis group in the two years prior to diagnosis (n, row %)

| **Healthcare contacts (0, 1-4, 5-9, 10+) by diagnosis group, per provider (n, row %)** | | | | | | | | | | | | | | | | | | | | | |
| --- | --- | --- | --- | --- | --- | --- | --- | --- | --- | --- | --- | --- | --- | --- | --- | --- | --- | --- | --- | --- | --- |
|  |  | *GP* | | | | | *Chiropractor* | | | | | *Physiotherapist* | | | | | *Medical specialist* | | | | |
|  |  | *0* | *1-4* | *5-9* | *10+* | ***Total*** | *0* | *1-4* | *5-9* | *10+* | ***Total*** | *0* | *1-4* | *5-9* | *10+* | ***Total*** | *0* | *1-4* | *5-9* | *10+* | ***Total*** |
| **Diagnoses group** | *Critical* | 41 (4.6%) | 136 (15%) | 227 (26%) | 478 (54%) | **882 (100%)** | 770 (87%) | 68 (7.7%) | 29 (3.3%) | 15 (1.7%) | **882 (100%)** | 659 (75%) | 164 (19%) | 37 (4.2%) | 22 (2.5%) | **882 (100%)** | 575 (65%) | 225 (26%) | 38 (4.3%) | 44 (5%) | **882 (100%)** |
|  | *Whiplash* | 715 (4.1%) | 3,451 (20%) | 4,672 (27%) | 8,705 (50%) | **17,543 (100%)** | 16,595 (95%) | 672 (3.8%) | 213 (1.2%) | 63 (0.4%) | **17,543 (100%)** | 16,117 (92%) | 1,098 (6.3%) | 231 (1.3%) | 97 (0.6%) | **17,543 (100%)** | 10,212 (58%) | 5,613 (32%) | 1,003 (5.7%) | 715 (4.1%) | **17,543 (100%)** |
|  | *Radiating* | 21 (3.5%) | 73 (12%) | 108 (18%) | 393 (66%) | **595 (100%)** | 439 (74%) | 88 (15%) | 51 (8.6%) | 17 (2.9%) | **595 (100%)** | 371 (62%) | 149 (25%) | 53 (8.9%) | 22 (3.7%) | **595 (100%)** | 377 (63%) | 166 (28%) | 30 (5%) | 22 (3.7%) | **595 (100%)** |
|  | *Structural* | 768 (5.6%) | 2,406 (18%) | 3,857 (28%) | 6,565 (48%) | **13,596 (100%)** | 12,443 (92%) | 749 (5.5%) | 314 (2.3%) | 90 (0.7%) | **13,596 (100%)** | 11,218 (83%) | 1,368 (10%) | 316 (2.3%) | 694 (5.1%) | **13,596 (100%)** | 9,200 (68%) | 3,327 (24%) | 577 (4.2%) | 492 (3.6%) | **13,596 (100%)** |
|  | *Regional* | 336 (2.4%) | 2,000 (14%) | 3,376 (24%) | 8,169 (59%) | **13,881 (100%)** | 11,950 (86%) | 1,221 (8.8%) | 525 (3.8%) | 185 (1.3%) | **13,881 (100%)** | 11,198 (81%) | 1,895 (14%) | 557 (4%) | 231 (1.7%) | **13,881 (100%)** | 8,265 (60%) | 4,134 (30%) | 793 (5.7%) | 689 (5%) | **13,881 (100%)** |
| **Total** |  | **1,881 (4%)** | **8,066 (17%)** | **12,240 (26%)** | **24,310 (52%)** | **46,497 (100%)** | **42,197 (91%)** | **2,798 (6%)** | **1,132 (2.4%)** | **370 (0.8%)** | **46,497 (100%)** | **39,563 (85%)** | **4,674 (10%)** | **1,194 (2.6%)** | **1,066 (2.3%)** | **46,497 (100%)** | **28,629 (62%)** | **13,465 (29%)** | **2,441 (5.2%)** | **1,962 (4.2%)** | **46,497 (100%)** |

Note: Values are presented as number (row percentage). Healthcare contacts are grouped into four categories (0, 1-4, 5-9, ≥10 contacts) for each provider type. Row percentages are calculated within each diagnosis group and provider type and sum to 100%. A “contact” refers to any reimbursed primary care service recorded in national registries; multiple contacts may occur within a single clinical encounter.
